# Antiretroviral treatment initiation does not differentially alter neurocognitive functioning over time in youth with behaviorally acquired HIV

**DOI:** 10.1007/s13365-015-0389-0

**Published:** 2015-10-13

**Authors:** Sharon L. Nichols, James Bethel, Bill G. Kapogiannis, Tiandong Li, Steven P. Woods, E. Doyle Patton, Weijia Ren, Sarah E. Thornton, Hanna O. Major-Wilson, Ana M. Puga, John W. Sleasman, Bret J. Rudy, Craig M. Wilson, Patricia A. Garvie, and the Adolescent Medicine Trials Network for HIV/AIDS Interventions

**Affiliations:** Department of Neurosciences, University of California, 9500 Gilman Drive, #0935, La Jolla, San Diego, CA 92093 USA; Westat, Rockville, MD USA; National Institutes of Health, Bethesda, MD USA; Department of Psychiatry, University of California, La Jolla, San Diego, CA USA; Children’s Diagnostic & Treatment Center, Inc., Ft. Lauderdale, FL USA; Department of Pediatrics, University of Miami, Miami, FL USA; Division of Allergy and Immunology, Duke University, Durham, NC USA; Department of Pediatrics, New York University, New York, NY USA; Department of Epidemiology, University of Alabama at Birmingham, Birmingham, AL USA

**Keywords:** HIV, Neurocognitive functioning, Adolescent, Youth, Antiretroviral therapy, HIV-associated neurocognitive disorder

## Abstract

Although youth living with behaviorally acquired HIV (YLWH) are at risk for cognitive impairments, the relationship of impairments to HIV and potential to improve with antiretroviral therapy (ART) are unclear. This prospective observational study was designed to examine the impact of initiation and timing of ART on neurocognitive functioning in YLWH in the Adolescent Medicine Trials Network for HIV/AIDS Interventions. Treatment naïve YLWH age 18–24 completed baseline and four additional assessments of attention/working memory, complex executive, and motor functioning over 3 years. Group 1 co-enrolled in an early ART initiation study and initiated ART at enrollment CD4 >350 (*n* = 56); group 2 had CD4 >350 and were not initiating ART (*n* = 66); group 3 initiated ART with CD4 <350 (*n* = 59) per standard of care treatment guidelines at the time. Treatment was de-intensified to boosted protease inhibitor monotherapy at 48 weeks for those in group 1 with suppressed viral load. Covariates included demographic, behavioral, and medical history variables. Analyses used hierarchical linear modeling. All groups showed improved performance with peak at 96 weeks in all three functional domains. Trajectories of change were not significantly associated with treatment, timing of treatment initiation, or ART de-intensification. Demographic variables and comorbidities were associated with baseline functioning but did not directly interact with change over time. In conclusion, YLWH showed improvement in neurocognitive functioning over time that may be related to practice effects and nonspecific impact of study participation. Neither improvement nor decline in functioning was associated with timing of ART initiation or therapy de-intensification.

## Introduction

Recent US guidelines call for all individuals with HIV infection to initiate antiretroviral therapy (Thompson et al. 2012) (ART) upon diagnosis, with prevention of central nervous system (CNS) sequelae serving as one argument for early initiation (Ellis et al. 2011). Despite ART having diminished the most severe CNS sequelae of HIV, subtle neurocognitive impairment continues to be a concern even for those on ART (Heaton et al. 2011). A meta-analysis found robust but modest positive effects of ART on HIV-associated cognitive dysfunction in adults (Al-Khindi et al. 2011). However, few studies have focused on individuals earlier in infection; while studies suggest a significant impact of acute HIV infection on the CNS is due to chronic inflammation that is independent of viral replication (Ancuta et al. 2008), it could be argued that control of viral replication by early ART may protect the CNS. Furthermore, late adolescents and young adults (hereafter referred to as “youth”) have received less focus in studies of ART and neurocognition, despite being a population at high risk for HIV and demonstrating relatively high rates of neurocognitive impairment (Nichols et al. 2013). Reservations regarding ART initiation in youth, including poor adherence resulting in emergence of drug resistant virus and toxicity associated with long-term treatment (Gagliardo et al. 2013; Lee et al. 2014), call for studies demonstrating neurocognitive benefit or at least lack of harm associated with ART in youth.

In 2007, the Adolescent Medicine Trials Network for HIV/AIDS Interventions (ATN) began enrollment for ATN 061, a prospective 3-year randomized clinical trial to study the immunological impact for youth of initiating highly active antiretroviral therapy (HAART) early (CD4+ T cell (CD4) counts >350 cells/mm^3^) compared to delaying treatment until concurrent treatment guidelines were met. This study also examined the effect of treatment de-intensification to boosted protease inhibitor (PI)-monotherapy among participants who achieved sustained viral suppression. The current study, ATN 071, was a 3-year prospective observational study designed to assess changes in neurocognitive functioning associated with early HAART initiation. The study included ATN 061 participants, additional untreated youth, and a group initiating ART per standard of care (SOC). Analysis of baseline data for ATN 071 demonstrated that 66 % of participants qualified for a diagnosis of HIV-associated neurocognitive disorder (Antinori et al. 2007) (HAND) at baseline, with impairments occurring most commonly in memory and learning, executive functioning, and fine motor speed (Nichols et al. 2013). Significant associations of some neurocognitive measures with CD4 count suggested possible subtle effects of HIV on neurocognition. Presented herein are longitudinal analyses of neurocognitive functioning over 3 years to determine whether ART, timing of ART initiation, and treatment de-intensification were associated with differences in the trajectory of neurocognitive functioning over time in ATN 071 while accounting for adherence and important confounding variables. The findings follow up the cohort described in Nichols et al. 2013 and represent the first study of the impact of ART on neurocognitive functioning among a key population affected by the HIV epidemic, late adolescents and emerging adults.

## Methods

### Participants

Youth aged 18–24 years with behaviorally acquired HIV infection were enrolled from 15 ATN and 5 International Maternal Pediatric Adolescent AIDS Clinical Trials sites across the USA and Puerto Rico. At the time of enrollment, the *US Department of Health and Human Services Guidelines for the Use of Antiretroviral Agents in Adults and Adolescents* (Guidelines) recommended starting ART in patients with CD4 cells <350, in absence of contraindications. Recruitment, participants, and assessment methodology are described in Nichols et al. 2013, with essential details repeated herein for convenience. Participants enrolled into four groups. Two groups had CD4 >350 at baseline: group 1 participants had been randomized to initiate early ART upon enrollment in ATN 061, while group 2 deferred ART until SOC guidelines for ART initiation were met. Group 3 participants met Guidelines for ART initiation with CD4 <350 at enrollment and started treatment. Group 4 (CD4 <350 and not initiating ART) was seen only at baseline and not discussed herein. Participants co-enrolled in ATN 061 were required to have HIV-1 RNA viral load (VL) >1000 copies/mm^3^. All participants were treatment-naïve except for ART to prevent mother-to-child HIV transmission (PMTCT; *n* = 3) with total duration of ART <6 months. Fluency in English or Spanish was required. Exclusion criteria included prior ART experience other than for PMTCT, current pregnancy, active substance use judged likely to interfere with meeting study requirements, psychosis, or significant non-HIV-related cognitive or motor impairment (e.g., cerebral palsy, severe traumatic brain injury; milder comorbidities including learning disabilities and attention-deficit/hyperactivity disorder were allowed). Participants who became pregnant while on study were discontinued due to potential impact of pregnancy on treatment considerations and cognition. The study was approved by Institutional Review Boards at all participating institutions; participants provided written informed consent in accordance with institutional requirements prior to enrollment.

### Study evaluations

#### Neurocognitive and behavioral functioning

Study design and evaluations are described elsewhere (Nichols et al. 2013). Participants were followed for 144 weeks, completing study evaluations at baseline and weeks 24, 48, 96, and 144.

The assessment battery included domains with previously demonstrated sensitivity to HAND in adults (Table 1), including memory, motor skills, attention, and complex executive functions. Standard scores using published normative data, with adjustments for age and, where available, race, Hispanic ethnicity, education and/or gender, were computed. Scores within domains were converted to *z*-scores and averaged for analytic clarity and to reduce the number of regression analyses performed. Neurocognitive measures were grouped into two sets: a monitoring battery conducted at all time points and a second set of measures administered only at baseline and exit (Table 1). Analyses reported herein focus on three scales derived from the monitoring battery reflecting attention, motor functioning, and complex executive functioning; individual tests were combined into domain scores to reduce the number of analyses required (Nichols et al. 2013). The domains included in the monitoring battery were selected based on anticipated sensitivity to change (Al-Khindi et al. 2011).

**Table 1:** Neurocognitive domains and assessment instruments

Domain of functioning	Test Name	Measure used in analyses
Monitoring battery (entry, wks 24, 48, 96, exit)		
Attention/working memory scale	Digit Span (WAIS-III; Psychological Corporation 1997)	Subtest standard score converted to z-score
	Letter-Number Sequencing (WAIS-III)	Subtest standard score converted to z-score
Motor/psychomotor scale	Digit Symbol (WAIS-III)	Subtest standard score converted to z-score
	Grooved Pegboard (Strauss et al. 2006)	Dominant hand total time z-score
	Timed Gait (Robertson et al. 2006)	Z-score for average time across 3 trials
Complex executive scale	Trailmaking Test (Part B; Reitan and Wolfson 1993; Mitrushina et al. 2005)	Total completion time z-score
	Controlled Oral Word Association (Strauss et al. 2006)	F, A, S & Animals total correct z-score
	Stroop Test (Interference trial; Norman et al. 2011; Golden and Freshwater 2002)	Interference trial z-score
Emotional/behavioral covariates	Beck Depression Inventory-II (Beck et al. 1987)	Total score
	Brief Symptom Inventory (Derogatis 1993)	Global Severity Index
	Alcohol, Smoking and Substance Involvement Screening Test (World Health Organization 2002)	Risk index for alcohol, marijuana, and other drugs
	Adherence	7-day self-report of percent doses taken
Entry and Exit only		
Global functioning	WAIS-III 5-subtest IQ estimate	Pro-rated IQ estimate derived from Vocabulary, Similarities, Block Design, Arithmetic, Matrix Reasoning subtest Scaled Scores
Learning and Memory	Hopkins Verbal Learning Test-R (Norman et al. 2011; Benedict et al. 1998)	Recognition and Recall corrected T- Scores
	Brief Visuospatial Memory Test-R (Norman et al. 2011; Benedict et al. 1996)	Recognition and Recall corrected T- Scores

*WAIS-III* Wechsler Adult Intelligenc Scale-Third Edition

Additional measures of depression, psychiatric distress, substance use, and medication adherence were administered for use as covariates in analyses. In addition, participants were asked whether they use potentially psychoactive substances (street drugs or medications; PPS), and whether they used them on the day of testing.

#### Demographics and psychosocial history

Participants reported birth sex, race, ethnicity, primary language, sexual orientation, employment, school enrollment status, past 30-day income, educational attainment, and educational risk (history of special education or repeating a grade).

#### Medical record abstraction

Comorbid current and past conditions were rated according to potential impact on current cognition as none, mild (e.g., headache, adjustment disorder), moderate (e.g., chronic migraines, major depressive disorder), or severe (e.g., seizure disorder, skull fracture), following published guidelines (Antinori et al. 2007). CD4 and plasma VL values within 4 weeks preceding the visit, CDC classification (1992), and date of first positive HIV test were abstracted. Treatment variables included concomitant medications, ART initiation, ART regimen, and, for group 1, de-intensification at ATN061 52-week time point following 24 weeks of sustained viral suppression.

### Statistical methods

#### Descriptive statistics

Treatment groups were compared using chi-square tests and *t* tests.

#### Modeling

Hierarchical linear modeling (HLM) using software HLM v7.0 (Raudenbush et al. 2011) was used to analyze the longitudinal neurocognitive scales. All scales (attention, motor, and complex executive) are expressed in *z*-score units with mean = 0. HLM accounts for the dependency in observations when data have a nested, multilevel structure, such as observations repeated at different times for the same study participant. The model also incorporates person-level covariates such as demographic characteristics or baseline clinical values. Included person-level baseline characteristics were group, gender, age, race/ethnicity, language spoken, education risk (special education class or repeated grade), education level, income earned in the past 30 days, CD4 count, time since HIV diagnosis, and viral load. Time-varying covariates included comorbid diagnosis rating; Beck Depression Inventory-2 (Beck et al. 1987) score; Brief Symptom Inventory (Derogatis 1993) score; medications with psychotropic effects; use of alcohol, cannabis, tobacco, or other drugs; and medication adherence (7-day self-report of missed doses). Finally, the model also included terms for status of anti-retroviral treatment (including early ART initiation) and ART de-intensification.

Growth curve modeling investigated whether there was nonlinear change in attention, motor, or complex executive indices over time. Both linear and quadratic time measures were included in the model (weeks and weeks^2^, respectively). Because of limits on the number of parameters that could be estimated with five time points and number of random effects, the linear and quadratic terms for time were estimated over all participants rather than for each individual. The growth curve models allowed for testing of group effects, demographic characteristics, and clinical covariates both as factors affecting overall neurocognitive performance and as interactions affecting changes over time in neurocognitive performance. Robust standard errors were used to account for non-normality (Liang and Zeger 1986). Follow-up analyses examined the effect of including baseline IQ in models to account for cognitive reserve; excluding individuals with severe comorbidities from models; and modeling treatment effects after grouping participants according to ART impact into those who never initiated ART (*n* = 36), those who initiated ART with consistent viral suppression across all subsequent time points (*n* = 56), and those who initiated ART with inconsistent or no viral suppression (*n* = 83).

## Results

### Population characteristics

Among 182 study participants enrolled between April 2008 and July 2010, baseline data were available for 181 participants. Participation dropped from 181 at baseline to 171, 151, 139, and 137 at weeks 24, 48, 96, and 144, respectively. Attrition was significantly higher in group 2 (*p* < 0.005); the most frequent reason was pregnancy, and participants who discontinued were significantly more likely than those who did not to be female (*p* = 0.006). Some individual test data were eliminated from analyses due to issues with validity (e.g., long fingernails interfering with fine motor performance).

Table 2 shows demographic characteristics of study participants at baseline. Mean age was 21.0 years. Participants were predominantly male (80.7 %), non-Hispanic Black/African-American (65.2 %) or Hispanic (22.7 %), self-identified as gay or bisexual (71.2 %), and high school educated or beyond (72.3 %), with 40.8 % currently in school and less than half (42.6 %) employed. Approximately 21.5 % reported having repeated a grade, and 22.7 % had received some form of special education. Nine participants (5 %) reported using a language other than English at home. Of those, one Spanish-speaking participant was tested in Spanish; the remainder, who were bilingual, completed testing in English. Demographic differences between groups are presented in Table 2.

**Table 2:** Demographic and baseline data

Characteristic	Group 1 initiating ART with CD4+ >350		Group 2, CD4+ >350, not initiating ART		Group 3 initiating ART with CD4+ <350		Overall		*p* value
	Count/mean	Percent/std. dev.	Count/mean	Percent/std. dev.	Count/mean	Percent/std. dev.	Count/mean	Percent/std. dev.	
Time point									
Baseline	56	100.0	66	100.0	59	100.0	181	100.0	0.0020
Week 24	52	92.9	63	95.5	56	94.9	171	94.5	
Week 48	48	85.7	53	80.3	50	84.7	151	83.4	
Week 96	48	85.7	45	68.2	46	78.0	139	76.8	
Week 144	47	83.9	44	66.7	46	78.0	137	75.7	
Age (years)	20.9	1.6	21.2	1.8	20.8	1.9	21.0	1.8	0.5104
Male gender	49	87.5	45	68.2	52	88.1	146	80.7	0.0051
Race/ethnicity									0.7933
Hispanic	11	19.6	18	27.3	12	20.3	41	22.7	
Non-Hispanic White	4	7.1	6	9.1	3	5.1	13	7.2	
Non-Hispanic Black/African American	38	67.9	38	57.6	42	71.2	118	65.2	
Asian/Pacific Islander	2	3.6	2	3.0	0	0.0	4	2.2	
Other or mixed race	1	1.8	2	3.0	2	3.4	5	2.8	
Language at home									0.4694
English	51	91.1	65	98.5	56	94.9	172	95.0	
Spanish	4	7.1	1	1.5	2	3.4	7	3.9	
Other	1	1.8	0	0.0	1	1.7	2	1.1	
Transgender	2	3.6	3	4.5	4	6.8	9	5.0	0.7661
Sexual orientation									0.0233
Straight (heterosexual)	10	17.9	25	37.9	11	18.6	46	25.4	
Gay/lesbian (homosexual)	41	73.2	31	47.0	39	66.1	111	61.3	
Bisexual	3	5.4	9	13.6	6	10.2	18	9.9	
Not sure/questioning	2	3.6	0	0.0	1	1.7	3	1.7	
Refused to answer	0	0.0	1	1.5	2	3.4	3	1.7	
Educational status									0.6315
Attending school	19	33.9	19	28.8	20	33.9	58	32.0	
GED program	5	8.9	4	6.1	7	11.9	16	8.8	
Not currently in school	30	53.6	42	63.6	32	54.2	104	57.5	
Other	2	3.6	1	1.5	0	0.0	3	1.7	
Level of education									0.8382
Eighth grade or less	2	3.6	1	1.5	2	3.4	5	2.8	
Not completed high school	16	28.6	17	25.8	12	20.3	45	24.9	
High school graduate	13	23.2	17	25.8	21	35.6	51	28.2	
Some education after high school	23	41.1	26	39.4	21	35.6	70	38.7	
College graduate	2	3.6	5	7.6	3	5.1	10	5.5	
Repeated a grade	14	25.0	10	15.2	15	25.4	39	21.5	0.3041
Special class or education	18	32.1	8	12.1	15	25.4	41	22.7	0.0283
Current employment									0.0098
Full-time	14	25.0	22	33.3	7	11.9	43	23.8	
Part-time	5	8.9	13	19.7	16	27.1	34	18.8	
Not employed	36	64.3	31	47.0	36	61.0	103	56.9	
Refused to answer	1	1.8	0	0.0	0	0.0	1	0.6	
Past year income									0.4411
<$6000	39	69.6	38	57.6	44	74.6	121	66.9	
$6000–$35,999	15	26.8	25	37.9	15	25.4	55	30.4	
Refused to answer	1	1.8	1	1.5	0	0.0	2	1.1	
Unknown	1	1.8	2	3.0	0	0.0	3	1.7	
Potential impact of comorbid diagnoses									
Severe	3	5.4	4	6.1	3	5.1	10	5.5	0.9894
Moderate	17	30.4	24	36.4	20	33.9	59	33.7	
Mild	5	8.9	7	10.6	6	10.2	16	10.0	
None	31	55.3	31	46.9	30	50.8	89	50.8	

### Clinical HIV characteristics

According to reported dates for first positive HIV test, approximately half the sample had been diagnosed with HIV for less than a year (Table 3; see Agwu et al. 2012 for discussion of length of infection in associated ATN 061). Significantly fewer participants in group 2 (7.6 %) had been diagnosed less than 4 months prior to baseline compared with group 1 (25.0 %) or group 3 (35.6 %) (*p* = 0.0024). Youth with CD4 counts >350 at baseline accounted for 94.6 % of group 1, 98.5 % of group 2, and 6.8 % of group 3; inclusion of participants in groups 1 and 2 with CD4 count <350 was due to changes in CD4 between eligibility screening and baseline assessments. Almost 90 % overall were in CDC category A, and all but 1.7 % had VL above 400 copies at baseline; 10.2 % of group 3 had AIDS by CD4 count <200 at baseline. Baseline VL distribution differed across the three groups, with 10.7 % of group 1 participants having VL above 100,000 copies vs. 3.0 % for group 2 and 17.6 % for group 3 (*p* < 0.005). ARV regimens were provider selected except for group 1, who initiated atazanavir/ritonavir plus tenofovir/emtricitabine as part of ATN 061. Regimens prescribed for group 3, as well as the subset of group 2 who subsequently initiated treatment per guidelines, were standard combinations of protease inhibitors (PI), nucleoside reverse transcriptase inhibitors (NRTI), non-nucleoside reverse transcriptase inhibitors (NNRTI), and integrase inhibitors (II). Altogether, 104 study participants used PI-based regimens during most or all of study duration, 34 were on NNRTIs, 2 were on II regimens, and 1 each were on NNRTI/II and PI/II regimens. Thirty-three participants used regimens containing efavirenz for most or all of study duration; an additional four used an efavirenz-containing regimen for part of their time on study. Reported adherence averaged 85 to 90 % over the course of the study (data not shown). There was a pronounced effect of treatment on both VL and CD4 counts, with VL decreasing and CD4 counts increasing between entry and week 144 for youth who initiated treatment (Table 3). Of group 1 participants, 35 underwent treatment de-intensification at week 48 to atazanavir/ritonavir monotherapy.

**Table 3:** Clinical data at baseline and end of study

Characteristic	Group 1 >350; starting ART		Group 2 >350; ART monitored per SOC		Group 3 <350; starting ART per SOC		Overall		*p* value
	Count/mean	Percent/std. dev.	Count/mean	Percent/std. dev.	Count/mean	Percent/std. dev.	Count/mean	Percent/std. dev.	
Time since HIV diagnosis at entry (months)									0.0024
<4	14	25.0	5	7.6	21	35.6	40	22.1	
4–11	18	32.1	17	25.8	19	32.2	54	29.8	
12–26	13	23.2	21	31.8	8	13.6	42	23.2	
≥27	11	19.6	23	34.8	11	18.6	45	24.9	
CDC classification for HIV at entry									0.0564
A	51	91.1	63	95.5	48	81.4	162	89.5	
B	5	8.9	3	4.5	10	16.9	18	9.9	
C	0	0.0	0	0.0	1	1.7	1	0.6	
Viral load									
At study entry									
<400	0	0.0	2	3.0	1	1.7	3	1.7	0.0049
400 to 10,000	17	30.4	36	54.5	15	25.4	68	37.6	
10,001 to 100,000	33	58.9	26	39.4	32	54.2	91	50.3	
100,001 to 500,000	5	8.9	2	3.0	10	16.9	17	9.4	
>500,000	1	1.8	0	0.0	1	1.7	2	1.1	
Mean (std. dev.)	4.3	0.5	3.9	0.6	4.4	0.7	4.2	0.6	0.0001
Median (range)	4.2	3.3–6.1	3.9	2.5–5.1	4.4	2.6–5.9	4.2	2.5–6.1	
At end of study									
<400	32	68.1	20	45.5	36	78.3	88	64.2	0.0117
400 to 10,000	9	19.1	12	27.3	3	6.5	24	17.5	
10,001 to 100,000	6	12.8	10	22.7	7	15.2	23	16.8	
100,001 to 500,000	0	0	2	4.5	0	0	2	1.5	
>500,000	0	0	0	0	0	0	0	0.0	
Meanlog_10_ units (std. dev.)	3.1	1.1	3.8	1.0	3.9	0.9	3.6	1.0	0.0631
Median (range)	3.4	1.4–4.7	3.9	1.6–5.7	4.2	2.1–5.0	4.0	1.4–5.7	
CD4+ count									
At study entry									
<200	0	0.0	0	0.0	6	10.2	6	3.3	<0.0001
200 to 349	3	5.4	1	1.5	49	83.1	53	29.3	
350 to 499	24	42.9	26	39.4	4	6.8	54	29.8	
≥500	29	51.8	39	59.1	0	0.0	68	37.6	
Mean (std. dev.)	552.4	188.4	595.6	200.6	275.7	79.0	478.0	218.1	<0.0001
Median (range)	506.5	280–1167	533.0	300–1160	288.0	16–498	440.0	16–1167	
At end of study									
<200	0	0.0	3	6.8	0	0	3	2.2	0.0025
200 to 349	3	6.4	4	9.1	9	19.6	16	11.7	
350 to 499	2	4.3	5	11.4	9	19.6	16	11.7	
≥500	42	89.4	31	70.5	25	54.3	98	71.5	
Mean (std. dev.)	808.8	278.6	584.5	225.5	556.1	212.6	654.6	266.2	0.0168
Median (range)	789.0	227–1476	604.0	80–1076	536.0	205–1067	651.0	80–1476	
CD4+ percent									
At study entry									
<15 %	3	5.4	0	0.0	18	30.5	21	11.6	<0.0001
15 to 24 %	17	30.4	23	34.8	37	62.7	77	42.5	
>24 %	36	64.3	43	65.2	4	6.8	83	45.9	
Mean (std. dev.)	27.2	7.1	28.3	8.2	17.5	6.2	23.8	8.7	<0.0001
Median (range)	28.4	13–40	28.1	15–50	18.0	1–34	23.8	1–50	
At end of study									
<15 %	4	8.5	5	11.4	2	4.3	11	8.0	0.4513
15 to 24 %	2	4.3	4	9.1	6	13	12	8.8	
>24 %	41	87.2	34	77.3	35	76.1	110	80.3	
Mean (std. dev.)	35.5	10.3	30.7	11.4	31.4	9.0	32.6	10.4	0.0168
Median (range)	38.0	10–53	32.0	0.3–52	31.0	7.5–50	34.0	0.3–53.0	

### Neurocognitive measures over time by group

**Fig. 1:**
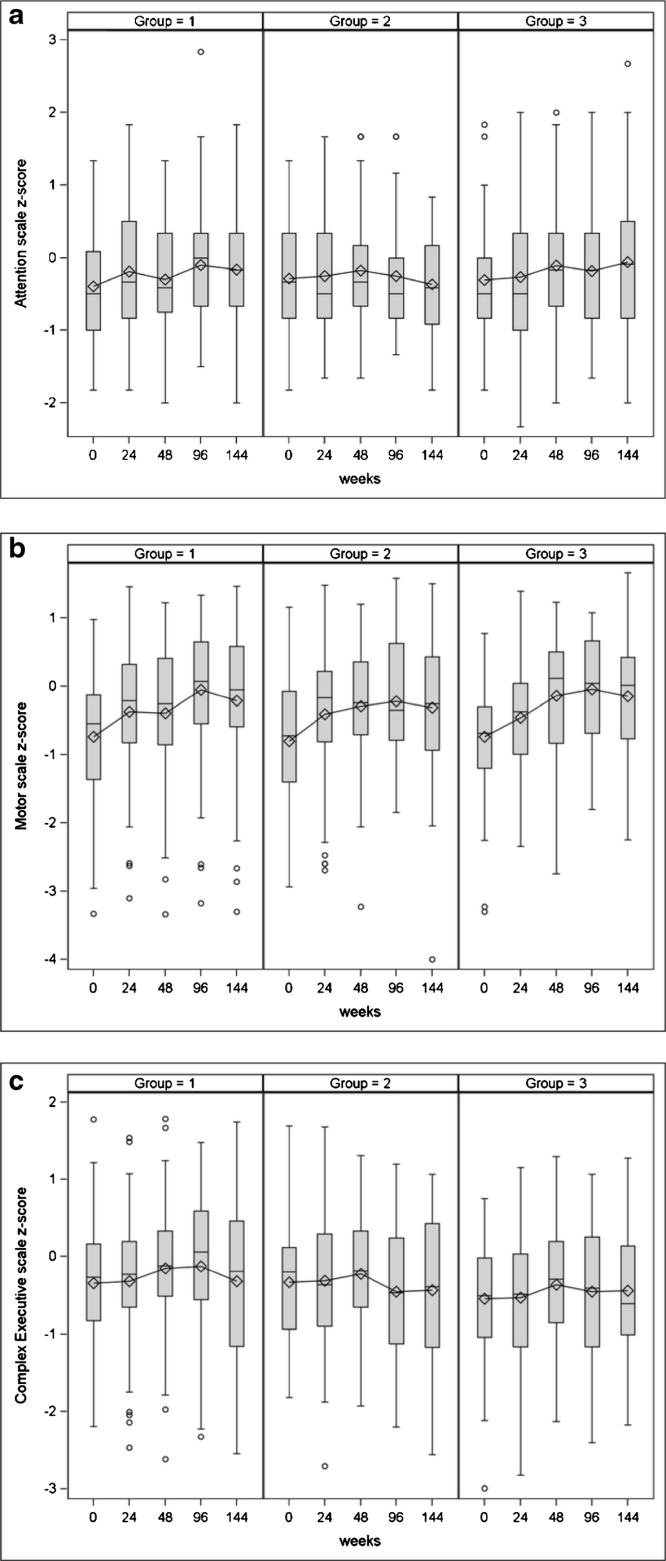
Descriptive statistics by group for **a** attention, **b** motor, and **c** complex executive scales over time

Figure 1a–c shows descriptive statistics by group for the three neurocognitive scales over time.

### Hierarchical linear modeling

Table 4 shows the results of fitting hierarchical linear models to the attention, motor, and complex executive scales. All models contain terms for demographic and clinical covariates, as well as for change over time.

**Table 4:** Hierarchical linear model for attention, motor and complex executive scales

HLM terms	Coefficient	Std. error	*p* value
Attention scale			
Model for baseline intercept			
Intercept	0.161	0.175	0.358
Hispanic (vs. non-Hispanic white and other)	−0.412	0.193	0.034
Non-Hispanic Black (vs. non-Hispanic white and other)	−0.349	0.178	0.052
Education risk (special class or repeated grade)	−0.427	0.106	<.001
Model for change over time			
Linear term	0.083	0.023	<.001
Quadratic term	−0.011	0.004	0.005
Effect of diagnoses with severe potential impact			
Intercept	−0.234	0.119	0.051
Baseline viral load	0.543	0.144	<.001
Effect of diagnoses with moderate potential impact			
Intercept	0.097	0.053	0.072
Baseline viral load	0.080	0.049	0.102
Motor Scale			
Model for baseline intercept			
Intercept	−1.045	0.153	<.001
Male gender	0.481	0.168	0.005
Model for change over time			
Linear term	0.304	0.027	<.001
Quadratic term	−0.037	0.004	<.001
Effect of diagnoses with severe potential impact			
Intercept	−1.670	0.195	<.001
Male gender	1.257	0.243	<.001
Effect of diagnoses with moderate potential impact			
Intercept	−0.474	0.244	0.053
Male gender	0.440	0.259	0.092
Complex executive scale			
Model for baseline intercept			
Intercept	0.179	0.127	0.161
Age	−0.081	0.033	0.016
Hispanic (vs. non-Hispanic White and other)	−0.575	0.138	<.001
Non-Hispanic Black (vs. non-Hispanic White and other)	−0.472	0.126	<.001
Education risk (special class or repeated grade)	−0.349	0.096	<.001
Model for change over time			
Linear term	0.089	0.025	<.001
Quadratic term	−0.014	0.004	0.001
Effect of diagnoses with severe potential impact			
Intercept	−1.640	0.119	<.001
Age	0.074	0.039	0.061
Male gender	1.529	0.121	<.001
CD4 at baseline	0.154	0.021	<.001
Viral load at baseline	0.702	0.068	<.001
Effect of diagnoses with moderate potential impact			
Intercept	−0.227	0.148	0.127
Age	−0.135	0.035	<.001
Male gender	0.377	0.160	0.020
CD4 at baseline	0.033	0.024	0.177
Viral load at baseline	−0.014	0.066	0.835

#### Attention index

The attention index, adjusted for covariates, increased from 0.161 at baseline to 0.317 at week 96, and then decreased slightly to 0.263 by week 144. Significantly lower attention index at baseline was seen in youth who were Hispanic, had education risk, or had comorbid diagnoses with potentially severe neurocognitive impact (e.g., seizure disorder). Other covariates (see “Methods” section) were not significantly associated with the attention scale. Covariates did not interact significantly with the attention index over time.

#### Motor index

The motor index increased from −1.045 at baseline to −0.421 at week 96 and then decreased slightly to −0.553 by week 144. Male youth had significantly better motor performance than females. Youth with comorbid diagnoses with potentially severe or moderate neurocognitive impact had significantly lower mean baseline motor index than those with mild or no diagnoses. Other covariates were not significantly associated with the motor scale.

#### Complex executive index

The complex executive index increased from 0.179 at baseline to 0.311 at week 96 and then decreased to 0.209 by week 144. Baseline executive index was significantly lower for participants who were older, Hispanic and non-Hispanic Black (vs. non-Hispanic White/other), had education risk, or had severe potential impact of diagnoses. Other covariates were not significantly associated with the complex executive scale.

Notably, there were no significant differences between study groups at baseline or in changes over time in the attention index, motor index, or executive index. Similarly, there were no observed differences in any index for participants who de-intensified their ARV medication. Relationships between covariates and index scores differed by comorbid diagnosis impact but did not interact with treatment (Table 4). Follow-up analyses showed that including baseline IQ as a covariate, excluding individuals with severe comorbidities, or grouping participants according to virologic response to ART did not cause a significant treatment effect on index scores to emerge. Higher full-scale IQ at baseline was associated with higher index scores at baseline and greater improvement in motor/psychomotor index scores over time, but the inclusion of IQ in models of group effects did not alter treatment findings.

## Discussion

This paper reports findings from the first prospective observational study of neurocognitive functioning in YLWH initiating ART at different CD4 thresholds. Models of trajectories of neurocognitive functioning over 3 years were examined for both positive and negative change. A broad neurocognitive, behavioral, demographic, and health history database allowed for the evaluation of several cognitive domains and multiple potential confounding factors. The key finding is that all three cognitive functional domains considered here improved significantly over time, each peaking at about 96 weeks into the study. Some degree of positive change was anticipated due to the likelihood of practice effects for neurocognitive measures (Duff 2012; Woods et al. 2006); our analytic approach was designed to compare the magnitude of change across groups. However, while there were modifying effects of demographic characteristics and non-HIV medical diagnoses, the magnitude and direction of change were not associated with treatment group or with clinical indicators of HIV-related disease factors. In addition, there were no differences associated with treatment de-intensification following viral suppression among early-treated youth, supporting safety of this management approach.

Prior studies with adults have found that ART is associated with modest improvements in cognitive functioning overall and that these improvements are seen largely in the areas of attentional, motor, and executive functioning (Al-Khindi et al. 2011). The sensitivity of these functional domains to HIV as well as to ART was the reason they were chosen for longitudinal monitoring in youth in the study reported here. The measures selected were ones that had a well-established track record in the adult HIV literature. The significant associations of the neurocognitive measures with other risk factors for the participants, such as educational risk and comorbid diagnoses, were in the predicted directions, suggesting that the lack of significant treatment effects was not likely due to insensitivity of the measures. Further support for measurement sensitivity comes from baseline findings showing subtle associations of some cognitive variables with CD4 count (Nichols et al. 2013). Nevertheless, treatment effects were not detected for our groups of youth with behaviorally acquired HIV.

There are several possible reasons that we did not observe effects of ART on cognitive functioning. One possibility is that, despite concern raised by the baseline findings regarding HIV impact on the CNS even in our relatively healthy cohort of youth, the contribution of HIV to neurocognitive functioning in our complex cohort is small enough relative to other influences and comorbidities that improvements with ART were too subtle to be detected with our sample size. A second possible explanation is that the medications did not adequately penetrate the central nervous system; a relatively narrow range of penetrance was represented and regimens for the most part were not highly penetrant (data not shown). A third possibility that could be raised is that participants were not adhering to their medications; however, reported adherence was reasonably good overall, supported by changes in CD4 and viral load, adherence was included as a covariate in analyses, and analyzing data according to virologic ART response did not alter the findings. Fourth, it may be that the observed impairments are related to persistent immune activation and inflammation established early in infection that is not reversed by ART (Ancuta et al. 2008; Wallet et al. 2010). Data collected from ATN 061 have demonstrated that T cell activation decreases with treatment but does not approach that of uninfected adolescents. In addition, ATN 061 demonstrated that, even with early initiation of ART, inflammatory markers including sCD14, sCD163, and sCD27 began and remained significantly higher than among a cohort of uninfected comparison youth similar in age, race, and substance use to the ATN 061 cohort (Rudy et al. 2014). An additional possibility is that the impairments seen at baseline were not related to HIV and that the CD4 findings were spurious. Rigorous measures were taken to account for other influences on neurocognitive functioning (Nichols et al. 2013), and exclusion of individuals with the most severe comorbidities did not affect the findings, but it should be acknowledged that controversy exists regarding the specificity of mild, asymptomatic neurocognitive impairments in adults with HIV disease (Gisslen et al. 2011; Grant et al. 2014; McDonnell et al. 2014). Further studies are needed to explore the impact of ART in the context of treatment begun in acute infection, using additional CNS measures such as neuroimaging, to evaluate regimens with a wider range of CNS penetrance, and to examine longitudinal associations with biomarkers of immune activation and inflammation.

With changes in US treatment guidelines, which now recommend ART in all HIV-infected persons regardless of immune function, evaluation of potential neurotoxicity of regimens has assumed greater importance. Particularly for young, asymptomatic individuals facing a lifetime of ART, concern about potential negative impacts of ART on functioning may decrease willingness to initiate or adhere to treatment. It is noteworthy that our findings indicated similar neurocognitive improvement in participants regardless of ART, suggesting lack of a negative impact of ART in general. Exploratory analyses including efavirenz, a medication that has been associated with CNS toxicity (Ciccarelli et al. 2011; Decloedt and Maartens 2013), did not find a differential effect on neurocognitive functioning. However, the number of youth taking this medication was small. Further studies are needed to support the safety of early ART in youth.

As noted in our description of baseline findings from ATN 071 (Nichols et al. 2013), a high percentage of youth in our cohort demonstrated neurocognitive impairment. Important questions remain for future studies, particularly if further studies continue to show that these impairments do not improve with ART. The origin of impairments may include modifiable factors such as substance use that could be the target of preventive interventions. Potential interventions could also include cognitive remediation, lifestyle modification such as exercise, as well as provision of enhanced education for healthcare-related and other daily living tasks for youth with impairment(s). Youth with the most significant impairments may be at risk for poor adherence, potentially leading to cognitive decline as part of disease progression (Ettenhofer et al. 2010); cognitive screening could be used to select youth for more intensive adherence support. The potential impact of impairments on other HIV prevention efforts, such as effectiveness of risk behavior reduction interventions, has public health implications and merits further research. Finally, long-term studies are needed to determine whether YLWH who have cognitive impairments are more vulnerable to cognitive decline associated with other risks such as head injury or aging. Exploratory analyses of our data demonstrated that neurocognitive impairment at baseline was associated with differences in trajectory of change, a finding that will be explored further in subsequent publications; however, it should be noted that inclusion of baseline cognitive performance or HAND diagnosis did not alter the finding of no treatment effects.

This study has several limitations. A primary limitation is that, while a portion of the participants with CD4 >350 were randomized to treatment or no treatment, the study as a whole was not a randomized clinical trial. In fact, treatment guidelines to initiate ART following HIV diagnosis preclude randomized studies comparing treated and untreated YLWH. Differences between the three study groups in some characteristics other than CD4 at baseline may have contributed to lack of a treatment effect and limit conclusions that can be drawn. However, it should be noted that analyses considered these characteristics as well as other variables. An additional limitation was the lack of an uninfected control group which could address nonspecific effects such as practice and possible developmental changes over time (however, age-standardized scores were used to account for development). Future studies should follow a well-matched uninfected group of youth to help account for these effects. Measurement of nonspecific clinical effects of participation, such as more frequent interaction with the clinic team, may also partially explain the non-ART-related positive change. Measures of memory and learning were included only at baseline and week 144 and were not included in these analyses; however, exploratory analyses demonstrated no associations of treatment with change in these measures. Although a significant number of youth were recently diagnosed with HIV, the study did not focus on youth with acute infection, a group likely to be most impacted by changes in treatment guidelines. Follow-up continued for 3 years, which may not have been protracted enough to observe longer term impacts of HIV and ART in youth. Finally, sample sizes were small for evaluation of some effects such as treatment de-intensification.

In conclusion, despite evidence of significant impairment at baseline, youth with behaviorally acquired HIV showed modest positive changes in neurocognitive functioning over 3 years of follow-up that were not related to antiretroviral treatment. Further research is needed to evaluate the impact of earlier treatment, newer regimens, and those with greater CNS penetrance.
